# Significant human health co-benefits of mitigating African emissions

**DOI:** 10.5194/acp-24-1025-2024

**Published:** 2024-01-24

**Authors:** Christopher D. Wells, Matthew Kasoar, Majid Ezzati, Apostolos Voulgarakis

**Affiliations:** 1 The Grantham Institute for Climate Change and the Environment, Imperial College London, London, UK; 2 School of Earth and Environment, University of Leeds, Leeds, UK; 3 Leverhulme Centre for Wildfires, Environment and Society, Department of Physics, Imperial College London, London, UK; 4 Department of Epidemiology and Biostatistics, School of Public Health, Imperial College London, London, UK; 5 MRC Centre for Environment and Health, School of Public Health, Imperial College London, London, UK; 6 Regional Institute for Population Studies, University of Ghana, Accra, Ghana; 7 School of Environmental Engineering, Technical University of Crete, Chania, Greece

## Abstract

Future African aerosol emissions, and therefore air pollution levels and health outcomes, are uncertain and understudied. Understanding the future health impacts of pollutant emissions from this region is crucial. Here, this research gap is addressed by studying the range in the future health impacts of aerosol emissions from Africa in the Shared Socioeconomic Pathway (SSP) scenarios, using the UK Earth System Model version 1 (UKESM1), along with human health concentration–response functions. The effects of Africa following a high-pollution aerosol pathway are studied relative to a low-pollution control, with experiments varying aerosol emissions from industry and biomass burning. Using present-day demographics, annual deaths within Africa attributable to ambient particulate matter are estimated to be lower by 150 000 (5th–95th confidence interval of 67 000–234 000) under stronger African aerosol mitigation by 2090, while those attributable to O_3_ are lower by 15 000 (5th–95th confidence interval of 9000–21 000). The particulate matter health benefits are realised predominantly within Africa, with the O_3_-driven benefits being more widespread – though still concentrated in Africa – due to the longer atmospheric lifetime of O_3_. These results demonstrate the important health co-benefits from future emission mitigation in Africa.

## 1 Introduction

Anthropogenic emissions of aerosols, their precursors, and reactive gases have substantial impacts on the climate. These impacts include a general aerosol cooling and, to a lesser extent, warming due to tropospheric O_3_ (Thornhill et al., 2021; Smith et al., 2020), as well as shifts in circulation patterns such as monsoons (Kasoar et al., 2018; Shawki et al., 2018; Wang et al., 2016; Liu et al., 2018). In addition to their climate effect, aerosols contribute to fine particulate matter air pollution, termed PM_2.5_ to denote particles with diameters less than 2.5 μm (Turnock et al., 2020). Reactive gases also modify concentrations of O_3_, which is another important climate forcer and air pollutant (von Schneidemesser et al., 2015). Due to their relatively short lifetimes, the effects of aerosols on the climate and human health depend on their emission location (Persad and Caldeira, 2018), with the health impact being particularly localised (Shindell et al., 2018). Since these species are co-emitted with greenhouse gas emissions, general climate change mitigation policies can lead to health co-benefits via reduced air pollution (Shindell et al., 2018).

The continent of Africa features a complex mix of air pollutant sources, both natural – such as dust from, for example, the Sahara – and anthropogenic, with continent-specific complexities such as imported second-hand high-emission vehicles (Abera et al., 2021). There is a broad range of possible future African pollutant emission pathways (Abera et al., 2021), some of which involve drastic increases in pollutants in key regions (Turnock et al., 2020). Recent and likely future dynamics of urbanisation can also be expected to drive enhanced exposure to air pollutants (Abera et al., 2021; Katoto et al., 2019). Reducing air pollution impacts in developing countries is a key component of the Sustainable Development Goals (Coker and Kizito, 2018).

The wide range of potential future African air pollutant emissions suggests concurrently disparate possible air pollution impacts over the continent. Despite this, studies assessing the human health impact of air pollution over Africa are sparse, especially those focused on outdoor air pollution (Abera et al., 2021), inhibiting the creation of appropriate concentration–response functions (CRFs) (Chen and Hoek, 2020; Abera et al., 2021; Katoto et al., 2019; Coker and Kizito, 2018). Observational data of air pollution in Africa are sparse (Coker and Kizito, 2018), with issues on data availability (Pinder et al., 2019) and structural barriers to reliable data collection (Pinder et al., 2019; Katoto et al., 2019). Strong intra-regional disparities in research persist, with two reviews finding over half of all studies measuring outdoor air pollution impacts in sub-Saharan Africa focusing on a single country (South Africa) and large areas entirely unstudied (Katoto et al., 2019; Coker and Kizito, 2018).

This study, while recognising the inadequacy of extant exposure research to effectively assess the effect of air pollutants in Africa, utilises recent CRFs for PM_2.5_ (GBD 2019 Risk Factors Collaborators, 2020), which incorporated high-air-pollution cohort studies from the Global South. This allows for a more accurate investigation of the effect of air pollution on human health in Africa than previously possible.

Global annual average PM_2.5_ concentrations have increased 15 %–20 % since the pre-industrial era to 6.9 ± 1.5 μg m^−3^ (Turnock et al., 2020) and are thought to still be slightly increasing in recent decades by 0.2 % yr^−1^, particularly over Asia and southern Africa (Gliß et al., 2021). The average concentration experienced by humans is much higher than this global average, due to the co-location of anthropogenic sources with population centres; 69 % of people are estimated to be exposed to PM_2.5_ concentrations higher than 10 μg m^−3^ (Lelieveld et al., 2013), with an average population-weighted PM_2.5_ exposure of 38 μg m^−3^, reducing to just 11 μg m^−3^ when excluding fossil fuel emissions (Vohra et al., 2021). The World Health Organisation recommends limiting long-term exposure to less than 5 μg m^−3^ (World Health Organisation, 2021), lowered in 2021 from their previous threshold of 10 μg m^−3^, though there is no known safe level of PM_2.5_ concentrations (Silva et al., 2013). Present-day human health impacts of PM_2.5_ are substantial but uncertain, with estimates varying from 2.37 (1.33–2.93) million deaths yr^−1^ (Partanen et al., 2018) to the more recent finding of 8.7 (−1.8 to 14.0) million deaths yr^−1^ (Vohra et al., 2021). In other studies, 3.61 (2.96–4.21) million deaths yr^−1^ have been attributed to fossil fuel PM_2.5_ alone (Lelieveld et al., 2019), and the Global Burden of Disease 2019 (hereafter GBD2019) estimated PM_2.5_-attributable deaths to be 4.14 (3.55–4.80) million deaths yr^−1^ (GBD 2019 Risk Factors Collaborators, 2020).

Anthropogenic activity has also increased annual average surface concentrations of O_3_, by 11.7 ppb (parts per billion) to the present-day levels of 29.9 ppb (Turnock et al., 2020). The lifetime of O_3_ is longer than that of PM_2.5_, but its effects are still strongly co-located with anthropogenic activity, with a population-weighted maximum 6-month average 1 h daily maximum concentration of around 57 ppb (Anenberg et al., 2010). Impacts of O_3_ on premature mortality have been estimated to be 0.7 ± 0.3 million deaths yr^−1^ (Anenberg et al., 2010) and 0.38 (0.12–0.73) million deaths yr^−1^ (Silva et al., 2016), using the same CRFs (Jerrett et al., 2009). Using more recent CRFs (Turner et al., 2016) 0.6 ± 0.1 million deaths yr^−1^ were attributed to O_3_-linked respiratory causes (Shindell et al., 2018), and GBD2019 attributed 0.365 (0.175–0.504) million deaths yr^−1^ to O_3_, purely from chronic obstructive pulmonary disease (COPD) (GBD 2019 Risk Factors Collaborators, 2020). Note that O_3_ concentrations presented in this study are annual average of the daily maximum 8 h mean concentration to be consistent with the CRFs used.

Future impacts of air pollutants will depend on both emissions and demographic changes. The reduced air pollution from measures targeting the direct lowering of carbon emissions rather than relying on negative emission technologies would prevent 93 ± 41 million deaths from PM_2.5_ and 60 ± 18 million deaths from O_3_ over the 21st century (Shindell et al., 2018). Measures compatible with 2 °C warming are projected to reduce life years lost due to PM_2.5_ by 0.7 million years per year in Europe by 2050 compared to a business-as-usual scenario, despite population increases (Schucht et al., 2015). Air pollutant emission decrease into the future in all the CMIP5-era Representative Concentration Pathway (RCP) scenarios, with reduced future air-pollution-linked mortality (Silva et al., 2016). However, the range in aerosol emissions between the different RCP scenarios is far smaller than that covered by the newer Shared Socioe-conomic Pathways (SSPs) (Gidden et al., 2019), which extend the RCP framework to include different socioeconomic and demographic trends and are projected to have substantially different air pollution impacts on human health (Im et al., 2023). Thus, the future range of possible human health impacts of air pollution is larger under more recent, less explored, scenarios.

Several previous studies have investigated the human health impacts of changes in global emissions but predominantly consider the global response. The effect of the newer SSPs on human health has also yet to be studied in detail with the latest CRFs.

The current study addresses these gaps using the SSPs to investigate the potential future impacts of African emissions on air pollution, both within and beyond the continent. Using the UK Earth System Model version 1 (UKESM1), a strong mitigation scenario was compared to three alternative emission scenarios, each substituting a subset of pollutant emissions over Africa for a weak mitigation equivalent.

## 2 Methods

### 2.1 Earth system model

This study uses the UK Earth System Model version 1 (UKESM1), a fully coupled global climate model used in the CMIP6 (Coupled Model Intercomparison Project phase 6) exercise. UKESM1 couples the ocean module NEMO (Nucleus for European Modelling of the Ocean) to its atmospheric module GA7.1 (Global Atmosphere 7.1) and the land module GL7 (Global Land version 7), with further couplings to Earth system components such as the biogeo-chemical scheme (Sellar et al., 2019). Its horizontal resolution is 1.875° × 1.25°, with 85 vertical levels. The atmo-spheric scheme features interactive chemistry, with 291 reactions and 84 species (Archibald et al., 2020). This is coupled to the Global Model of Aerosol Processes (GLOMAP)-mode aerosol scheme, which simulates the concentrations of black carbon (BC), organic carbon (OC), sulfate, sea salt, primary marine organic aerosol (PMOA), and secondary organic aerosol (SOA) in five lognormal modes in total, with four soluble and one insoluble (Bellouin et al., 2013; Mulcahy et al., 2020). The GLOMAP mode is a two-moment scheme, calculating both aerosol mass and number concentration, allowing different processes to impact these independently; it simulates both aerosol direct and indirect effects (Mann et al., 2010), with broader semi-direct effects enabled via the coupling to the dynamical atmosphere. Dust is treated separately within UKESM1 via the older one-moment (mass only) CLASSIC scheme (Bellouin et al., 2011).

UKESM1’s representation of surface PM_2.5_ and O_3_ has been evaluated in relation to observations and other models (Turnock et al., 2020; in particular, their Figs. 3–8). In areas that are well sampled with surface PM_2.5_ measurements, UKESM1 is consistent with other CMIP6 models, exhibiting a low bias in PM_2.5_ in Eastern Europe and North America by around 2–10 μg m^−3^. Over oceans, PM_2.5_ is also systematically low, but the picture over other land areas is mixed when compared to MERRA (Modern-Era Retrospective Analysis for Research and Applications) reanalysis. In the multi-model mean, PM_2.5_ concentrations over northwest Africa are too low (Turnock et al., 2020), while those over eastern and southern Africa are too high by around 2–15 μg m^−3^ between models. Concentrations over Asia are also generally too high, with all bias patterns roughly similar between December to February (DJF) and June to August (JJA). UKESM1 is typical in its PM_2.5_ bias across most regions, including northern Africa. In sub-Saharan Africa, however, it exhibits a stronger seasonal cycle than other models, with the main biomass burning seasons featuring substantially higher PM_2.5_ concentrations than other models and the observational best estimate. Simulated PM_2.5_ concentrations are up to 50 % higher than the multi-model and observational means in July and January, though they still lie close to the wide observational range. The areas of high biases in CMIP6 are areas with high background PM_2.5_ and large ranges in simulated concentrations across CMIP6, with inter-model standard deviations of over 20 μg m^−3^ in the most polluted areas of northern and central Africa.

CMIP6 models generally exhibit high biases in surface O_3_, overestimating North American, European, and East Asian concentrations by around 10 ppb in DJF and JJA compared to surface observations (Turnock et al., 2020). UKESM1 has typical biases in JJA (i.e. high) but overestimates the amplitude of the seasonal cycle, becoming the only one of five CMIP6 models studied by Turnock et al. (2020) to exhibit a low bias over northern hemispheric land. As for PM_2.5_, the areas with the largest concentrations and inter-model standard deviations are the high-emission regions in Africa and Asia. UKESM1’s representation of O_3_ over sub-Saharan Africa is much closer to the multi-model mean; the lack of local surface observations precludes a full evaluation, though the sole observational station in South Africa closely tracks the model’s regional averages (Fig. 4 in Turnock et al., 2020). Aerosol optical depth (AOD) in UKESM1 is consistent with satellite observations in low-AOD areas but is biased low over some areas with strong aerosol emissions such as West Africa (Mulcahy et al., 2020).

### 2.2 Experiments

This study uses the Shared Socioeconomic Pathway (SSP) emission trajectories to estimate the future health impact of different African emission pathways. The SSPs are denoted SSP*x* − *y*, with *x* being an integer referring to one of five socioeconomic narrative pathways to explore different non-climate societal evolutions, and *y* denoting the top-of-atmosphere (TOA) radiative forcing in 2100 under a particular mitigation scenario (O’Neill et al., 2017). The SSPs there-fore explore a range of future possible trajectories covering both socioeconomic and mitigation trends. This project uses SSP119 as a control scenario. Designed to be roughly consistent with strong mitigation under the Paris Agreement, this follows socioeconomic trajectory 1 – “sustainability” (van Vuuren et al., 2017) – along with broad emission reductions to approximately reach 1.9 W m^−2^ radiative forcing in 2100. To test the effect of weaker mitigation in Africa for different sets of emissions, three experiments are simulated, switching out the SSP119 aerosol and reactive gases emissions over Africa for their SSP370 equivalent. SSP370 follows the so-cioeconomic trends in narrative 3 – “regional rivalry” (Fujimori et al., 2017) – coupled with weak mitigation, leading to a TOA radiative forcing of around 7 W m^−2^ in 2100. The three experiments performed are named after the SSP370 emission subset which is substituted over Africa; the full set of experiments is as follows:

Control, with SSP119 globally;

AerAll, which is the control with African aerosol emissions from SSP370;

AerNonBB, which is the control with African non-biomass burning aerosol emissions from SSP370;

AerBB, which is the control with African biomass burning aerosol emissions from SSP370.

AerAll indicates that all aerosol and reactive gas emissions over Africa are substituted with SSP370, while emissions over all other areas, and for other climate forcers such as well-mixed greenhouse gases (GHGs) over Africa, are kept at their SSP119 values as in the control. AerBB then switches out just the biomass burning (BB) components of aerosols and reactive gases, and AerNonBB changes only the non-BB emissions (i.e. fossil fuel and biofuel) over Africa. Note that the terminology of non-BB is used in this paper to refer to the non-biomass-burning emissions themselves (i.e. those from fossil fuels and biofuels), which are changed in both AerAll and AerNonBB, and so the effects of changed non-BB emissions are found under both experiments. BB, similarly, refers to biomass burning emissions that changed in both AerAll and AerBB. Non-BB emissions are purely anthropogenic, while those from biomass burning are complexly related to human activity, particularly over Africa (Bauer et al., 2019), driving the counter-intuitive increase in BB emissions in the stronger mitigation scenario. Pollutant concentrations are not bias-corrected here, in order to determine the specific estimation in UKESM1 and due to the sparse observations over Africa. The focus is therefore on the relative impact of the scenarios, while also contextualising the magnitudes in relation to prior studies.

The control scenario thus depicts a global future with strong climate mitigation policies, leading to relatively low greenhouse gas emissions and consequently low-air-pollutant levels. Each of the alternative scenarios represents a future in which Africa instead follows a more “pessimistic” scenario in its emissions of air pollutant precursors, allowing for an exploration of the health impacts of such a range in future trajectories.

The aerosol emissions changed are BC and OC, and the reactive gases are C_2_H_6_, C_3_H_8_, CO, dimethyl sulfide (DMS), HCHO, Me_2_CO, MeCHO, NH_3_, NO, lumped non-methane volatile organic compounds (NVOCs), and SO_2_. In UKESM1, all of these emission species have both BB and non-BB components, except for SO_2_, which has only a non-BB component. All are emitted from the surface, except a subset of BB BC and OC representing large fires, which are injected vertically and uniformly from the surface to 3 km, and aircraft NO emissions, which are injected in a 3D grid. It should be noted that, since the aerosol and O_3_ precursors are co-emitted with greenhouse gases, these scenarios changing emission subsets are not realistic future scenarios. Instead, the purpose is to investigate the range of plausible human health impacts between scenarios, which are driven by the species altered in these experiments.

Multiple ensemble members were simulated for each experiment; each was initiated in 2015 with slightly different atmospheric and ocean conditions to explore the internal climate variability. There are 10 ensemble members of SSP119 used for the control – five simulated for this study and five taken from the UKESM1 CMIP6 experiments (Tebaldi et al., 2021) – and seven of each of the other experiments are simulated. All simulations run the length of the SSP scenarios, i.e. 2015–2100. The analysis of the health impacts here focuses on the effects in 2090. For the O_3_ impacts, the five UKESM1 CMIP6 control members did not output the concentrations hourly, so only the five control experiments simulated for this project were used for the control concentrations. The local and remote climate impacts of these emission scenarios, plus additional scenarios changing CO_2_ emissions in a similar manner, are explored in a separate paper (Wells et al., 2023).

Figure S1 in the Supplement indicates the time evolution of the aerosol and SO_2_ emissions over Africa and globally in the control (black) and experiments (red), with the total and BB carbonaceous aerosol shown. Also shown is maps of the emission differences for carbonaceous aerosol and SO_2_. Total carbonaceous aerosol emissions over Africa decline quickly in the SSP119 control, consistent with general emission mitigation, whereas they remain roughly flat in SSP370. This acts to dampen the general global decrease in emissions, though they still almost halve at the global level through the 21st century as the rest of the world follows SSP119. The BB emission subset, however, shows the opposite (and weaker) trend, with emissions remaining approximately constant in SSP119 but declining in SSP370, while global emission decline in each case. This is inconsistent with the general emission reductions in SSP119 and is reflective of the more complex link between anthropogenic activity and BB emissions than between human actions and non-BB emissions. Different IAMs (integrated assessment models) were used to produce the emission pathways for the different scenarios (IMAGE for SSP1 and AIM/CGE for SSP3; Fujimori et al., 2017; van Vuuren et al., 2017); this makes a clear understanding of the differences between complex emission sources difficult, but it likely relates to different land use activity in the scenarios. Non-BB emissions still dominate the carbonaceous aerosol emission change, as indicated by the larger overall carbonaceous emissions in SSP370 than SSP119 over Africa. The SO_2_ emission change features a complex pattern, with emissions higher across most of the continent in SSP370 than SSP119 but relatively lower over southern Africa (except South Africa). In both cases, emissions drop substantially overall, and the differing spatial changes over Africa approximately cancel, resulting in little overall emission difference between the scenarios. As with the BB aerosol changes, the specific cause of the differing trends in SO_2_ emissions is hard to discern, though it is driven by stronger industrial SO_2_ emissions in SSP119 (Gidden et al., 2018), indicating a projected faster industrialisation in SSP119 than SSP370 in southern Africa.

### 2.3 Health impact analysis

Many studies utilise a common methodology to estimate the human health impact of a given concentration, or change in concentration, of pollutants (e.g. Anenberg et al., 2010; Shindell et al., 2018). Cohort studies, tracking a large population over many years, are used to produce empirically determined concentration response functions (CRFs), linking background air pollutant concentrations to the change in the relative risk (RR) of dying from a particular cause of death (COD). The RR at 0 concentration is 1 by definition and increases monotonically above a low-concentration threshold (LCT). The form of RR is constrained by the fit used to derive the function. Early studies used exponential fits (Pope et al., 2002), while others use linear relationships or power laws (Pope et al., 2009; Ezzati et al., 2004), while more recent studies use more complex functional forms (Burnett et al., 2014, 2018).

While various co-founding factors are controlled for – such as lifestyle and income level – it is not necessarily valid to generalise a RR from a single cohort to the global scale. This issue especially applies to the extrapolation of pollutant concentrations to levels outside those experienced by the cohort population. In particular, a large American Cancer Society cohort study was used to generate earlier RR curves, but the highest PM_2.5_ concentrations that this cohort was exposed to were less than 30 μg m^−3^, lower than the global population-weighted average of 38 μg m^−3^, as found by Vohra et al. (2021). This gap can be bridged using data from active smoking, but this assumes that a short, high-exposure burst – from smoking individual cigarettes – has the same health effect as a lower, continuous background concentration (Smith and Peel, 2010; Pope et al., 2009). More recent studies use multiple cohort studies across a range of ambient exposures, significantly mitigating this issue (Burnett et al., 2018; GBD 2019 Risk Factors Collaborators, 2020) and rendering such CRFs more applicable to highly polluted regions than prior estimates. At the other end of the exposure range, the assumed LCT below which the RR is 1 (i.e. pollutant concentrations below this have no human health effect) has decreased in consecutive studies, as cohorts in evercleaner environments still exhibit significant effects of air pollution; there is no biological justification for a threshold, and more recent CRFs, including that used here, use a statistical distribution to represent the LCT (GBD 2019 Risk Factors Collaborators, 2020).

The attributable fraction (AF) estimates the fraction of deaths – of a particular COD – attributable to the air pollutant exposure as follows (Mansournia and Altman, 2018):

(1) AF=(RR−1)/RR=1−(1/RR)

Given a COD-specific RR curve, and common grids of surface concentrations of the pollutant (either PM_2.5_ or O_3_), baseline population (Pop), and mortality for a specific COD (*y*_0_), the number of deaths attributable to the pollutant can be estimated as

(2) $$\text{Deaths}\;=y_{0}\cdot \text{Pop}\cdot \text{AF}$$

Equation (2) is applied at each grid cell to determine the estimated annual deaths within the cell.

The concentration–response functions used in this study are taken from the Global Burden of Disease 2019 (hereafter GBD2019; GBD 2019 Risk Factors Collaborators, 2020) for PM_2.5_ and from Turner et al. (2016) for O_3_. While studies prior to GBD2019 imposed functional forms of varying complexity on their CRFs, GBD2019 uses a Bayesian metaregression method to provide the fit, with only the assumption that the CRF should be monotonic. Due to the uncertainties regarding the existence and level of safe low concentrations of PM_2.5_, GBD2019 suggests the use of a uniform distribution from 2.4–5.9 μg m^−3^ for the LCT, representing the lowest and the 5th percentile concentrations found in the background concentrations; this threshold is used in this study. The GBD2019 dataset provides 1000 draws of the fit for each COD–age pair with no threshold; an LCT from the suggested uniform distribution was then randomly selected for each draw to complete the distribution. These 1000 draws represent the uncertainty in the CRF; the median, 5th, and 95th percentile impacts using these draws are calculated here to explore this uncertainty.

GBD2019 provides CRFs for six COD for PM_2.5_: lung cancer (LC), chronic obstructive pulmonary disease (COPD), lower respiratory infection (LRI), type-2 diabetes (T2DM), stroke, and ischemic heart disease (IHD). The latter two are age-dependent on 5-year brackets; T2DM applies only to populations over 25 years; and the other COD are applied to the total population. The CRFs for PM_2.5_ apply to annual average PM_2.5_ concentrations.

Following prior studies (Malley et al., 2017; Shindell et al., 2018; GBD 2019 Risk Factors Collaborators, 2020), the CRF for O_3_ in this study was taken from Turner et al. (2016) for respiratory mortality, which includes COPD, LRI, upper respiratory infections, asthma, pneumoconiosis, interstitial lung disease, pulmonary sarcoidosis, and other chronic respiratory diseases. This CRF applies to populations over 30 years of age and applies to the annual average of the daily maximum 8 h mean concentration. An LCT of 26.7 ppb, the minimum concentration found in the cohort studies used by Turner et al. (2016), is applied here, with a sensitivity test carried out applying an LCT of 31.1 ppb, representing the 5th percentile in the underlying cohort data.

This study uses the following approximation for PM_2.5_:

(3) $${\text{PM}}_{25}=\text{OC}+\text{BC}+{\text{SO}}_{4}+0.25\cdot \text{SS}+0.1\cdot \;\text{dust}.$$

This means that all carbonaceous and sulfate aerosol contributes to PM_2.5_, but only 25 % of sea salt (SS) and 10 % of dust are assumed to be shorter than 2.5 μm in diameter. This approximation is used in AerChemMIP (Turnock et al., 2020) and other studies (e.g. Allen et al., 2021).

Single years of pollutant concentrations, averaged across the ensembles, were utilised in this study, namely 2015, 2050, and 2090. There are several reasons for the choice to use single years rather than, for example, averaging over a decade to smooth out interannual variability. The present-day value needed to be centred around 2015, at the start of the scenarios, since this is where the emissions start to diverge. All simulations were initialised from 2015, so it would not have been possible to use a larger window to average around 2015. If data past 2015 had been used, e.g. 2015–2025 for the present day, then this would have introduced other issues. Since the emission scenarios diverge from 2015, the choice of scenario to take the data from would affect the results, and this would not represent the present-day in the other scenarios; in addition, the rapid decrease in emissions from 2015 in all scenarios would mean the 2015–2025 average would be significantly lower than the concentrations in 2015 and therefore not closely represent the conditions experienced by the 2015 population distribution under present-day concentrations.

Population numbers from the SSPs in 2015 – equal between scenarios since the SSPs only diverge after 2015 – were used to ensure consistency across the methodology (Lutz et al., 2018). Equation (2) is applied in each model grid cell level, using the pollutant concentrations output from UKESM1, so the country level population data were re-gridded to the 1.875° × 1.25° UKESM1 grid. To approximately preserve present-day within-country population distributions, a high-resolution (0.25° × 0.25°) present-day population file was used (CIESIN, 2018), and a country name was assigned to each cell within this grid using a global shapefile (Sandvik, 2008). The present-day population distribution within each country was then scaled to create the correct total for each age–year pair, and these distributions were then re-gridded to the UKESM1 grid resolution. Baseline mortality data for each COD–age pair were applied at the country level (IHME, 2020) and re-gridded to the UKESM1 grid using the global shapefile.

Present-day populations were used for the analysis for two reasons. First, this was done to isolate the effect of changes in emissions on human health. Second, while the SSPs include population projections, they do not include future baseline mortality estimates, and present-day mortality rates cannot be assumed constant while populations and other social factors change significantly and differently between scenarios.

## 3 Results

### 3.1 Air pollution impact

Africa is a continent with a major presence of key pollutants compared to the global average, as seen in Fig. S2. Based on our UKESM1 simulations, the organic carbon (OC) contribution to PM_2.5_ is generally highest in the tropical biomass burning regions, peaking in Africa; this is also true for atmospheric dust. The distribution of O_3_ is smoother than that of PM_2.5_, owing to its longer lifetime, with the concentrations again being higher than average near the main emission regions in the low latitudes in Africa.

**Figure 1:**
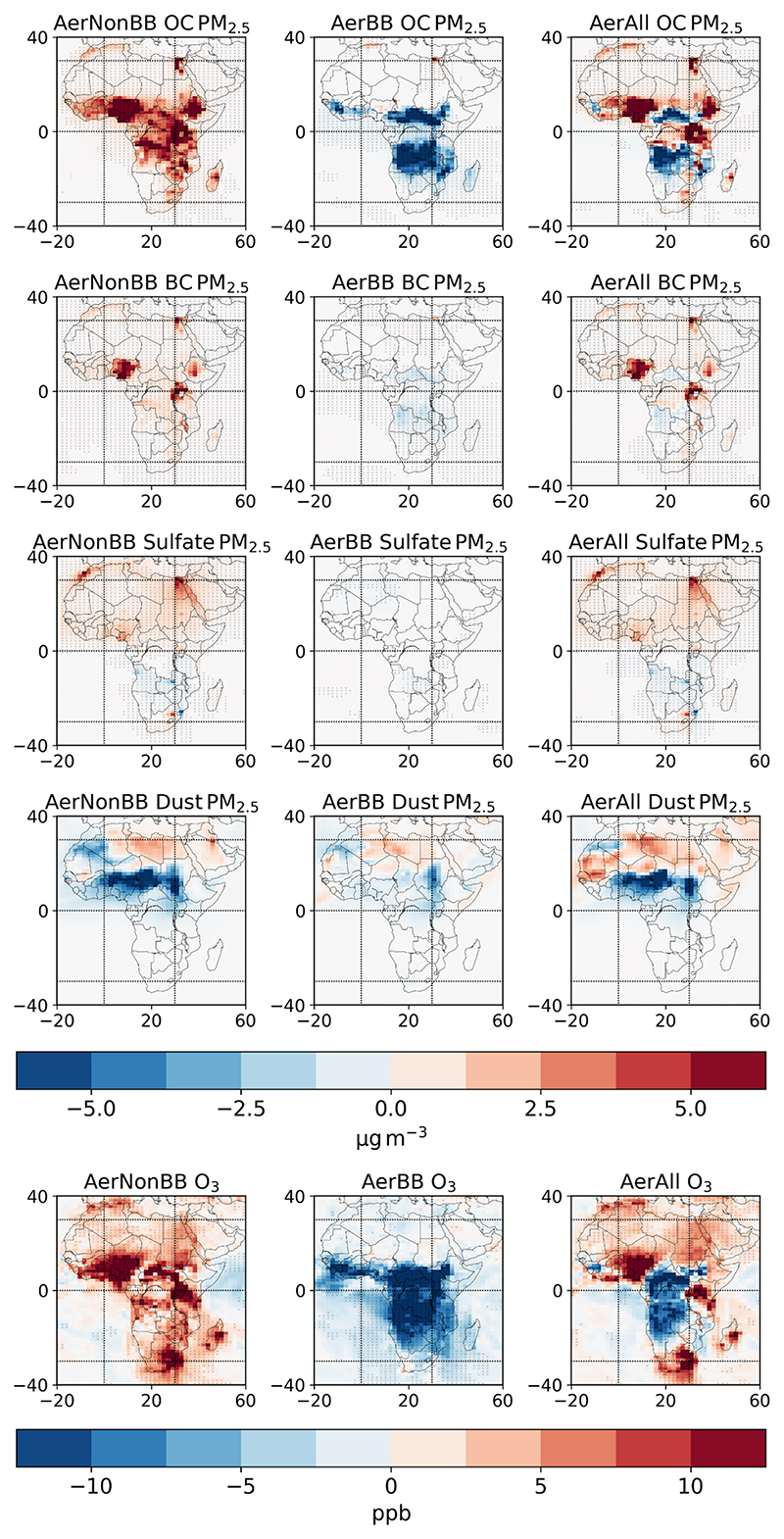
The change in the organic carbon (OC), black carbon (BC), sulfate, and dust aerosol contributions to surface PM_2.5_, and O_3_, under each emission scenario relative to the SSP119 control in 2090 over Africa. Stippling indicates areas where the ensemble mean change is greater than 1 intra-ensemble standard deviation away from 0.

The changes in surface PM_2.5_ and O_3_ over Africa near the end of the century (2090) for all simulations, split into contributions from each component, are shown in Fig. 1. Figure S3 shows the corresponding time series of simulated pollutants for Africa, its sub-region West Africa, and the neighbouring region Europe, as well as for the whole globe. The simulations explore the change in pollution levels in scenarios where the whole globe follows a strong mitigation pathway, while Africa follows a more pessimistic policy pathway in terms of its biomass burning aerosols (AerBB simulation; though, as discussed in Sect. 2, these emissions are higher in the control), non-biomass burning aerosols (AerNonBB), and all aerosols combined (AerAll) (see Sect. 2). The carbonaceous aerosol increases under AerNonBB are near the main non-biomass burning emission regions, especially in West and East Africa, while reductions under AerBB are centred on the biomass burning regions north and south of the Equator. AerAll then exhibits features of both. Sulfate shows weaker changes, with the North African non-BB increased emissions, contrasting with the reduced emissions south of the Equator. Changes in dust are significant, with a substantial decrease in areas with high background dust emissions. This decrease in dust emissions is ultimately due to the impact of the aerosol emissions on local surface winds, which drives the emission of dust (see Fig. S4). O_3_ follows a similar pattern to the PM_2.5_ changes, as its concentration is modified by the reactive gases co-emitted with the anthropogenic aerosol species.

### 3.2 Health impact

**Figure 2:**
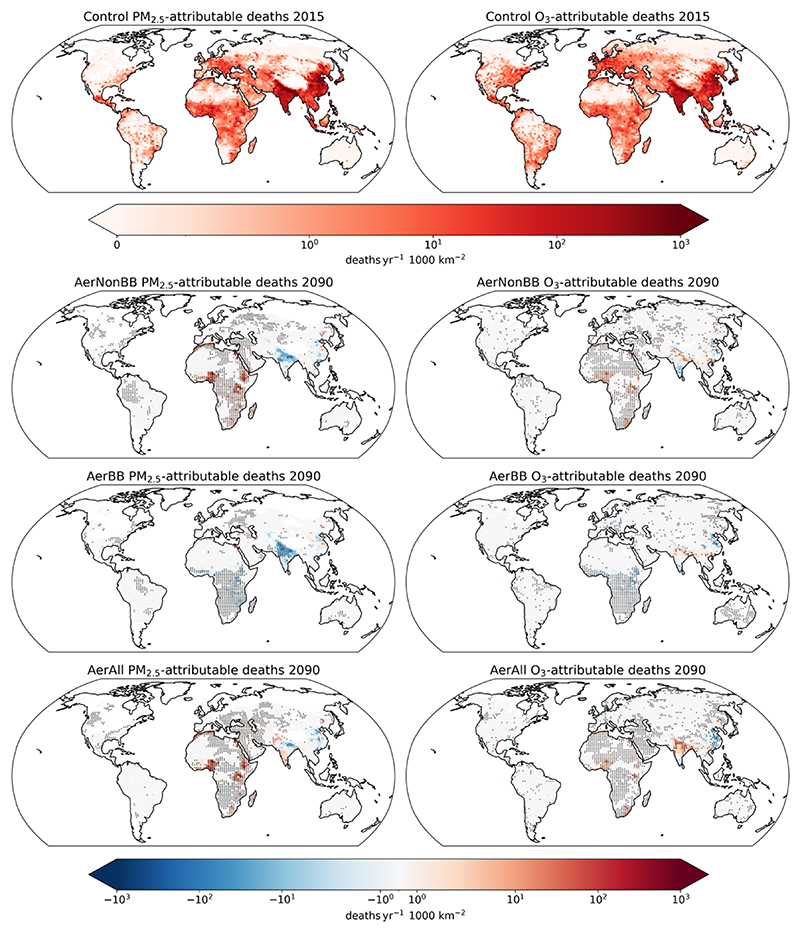
Top row shows the annual deaths per 1000 km^2^ attributable to air pollution for all COD for PM_2.5_ and respiratory illnesses for O_3_ in 2015. Subsequent rows show the impact of each scenario on 2090 deaths per 1000 km^2^ attributable to PM_2.5_ and O_3_ exposure relative to the SSP119 control, using 2015 spatial and age-based population distributions. Stippling indicates areas where the change is greater than 1 intra-ensemble standard deviation away from 0.

The estimated deaths in 2015 from PM_2.5_ (six causes of death (COD); see Sect. 2) and O_3_ (respiratory illnesses only) per 1000 km^2^ are shown in the top row of Fig. 2. Table 1 indicates global and regional totals. The control SSP119 experiment is used for the air pollutant concentrations in 2015, which is an arbitrary choice as the SSP emissions diverge only after this year.

Globally, 2.76 (2.11–3.48) million annual deaths are attributed to PM_2.5_, with ischemic heart disease (IHD) being the largest COD, followed by stroke, and 2.28 (1.73–2.70) million to O_3_. Deaths are highest in areas where population densities and pollutant concentrations are high, namely in East and South Asia and in tropical Africa. The highest number of deaths attributable to PM_2.5_ and O_3_ exposure occur in Asia; Africa experiences large impacts too, with 12 % and 7.4 % of the global total for PM_2.5_ and O_3_, respectively.

The second to fourth rows in Fig. 2 show the effect on annual deaths per 1000 km^2^ in 2090 of the different emission scenarios, relative to the SSP119 control scenario, for both PM_2.5_ and O_3_. Population distributions from 2015 are used here to isolate the effect of the changed pollutants. The spatial pattern of human health impacts is broadly consistent with the emission changes, modulated by the population distribution. AerNonBB features higher deaths across Africa than in the control, particularly in the highly populated west and east regions, due to future increases in fossil fuel emissions. The decrease in dust emissions in the southern Sahara leads to an overall reduction in PM_2.5_ – and therefore lower health impacts – in this region (see Fig. S4). This indicates that the indirect effects of pollutant emissions on atmospheric circulation – and therefore natural dust emissions – can have a substantial influence on their overall impact, as also noted by, for example, Bauer et al. (2019) and Yang et al. (2017). The lower future African biomass burning emissions in the AerBB experiment compared to the control result in significantly lower deaths across central and southern Africa. Still, the co-location of fossil fuel emissions with population centres causes these emissions to dominate the overall impact in AerAll.

**Figure 3:**
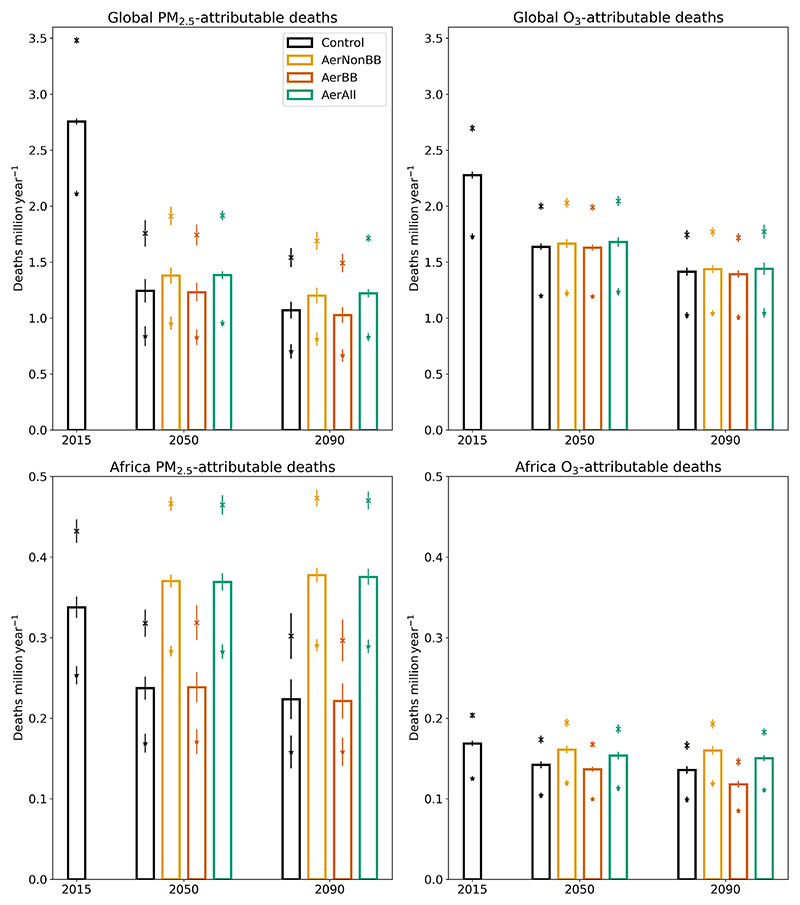
Annual PM_2.5_- and O_3_-attributable deaths in the control in 2015 and each scenario in 2050 and 2090, globally and over Africa, using 2015 spatial and age-based population distributions. The bars indicate the estimated deaths using the central CRF estimates; the crosses and stars use the 95th and 5th percentile CRF values respectively. For each CRF value, the (much smaller) uncertainty due to intra-ensemble variation in pollutant concentrations is indicated with vertical error bars. This intra-ensemble variation is defined as the estimated 5th–95th percentile range across the 10 control ensemble members, calculated as the standard deviation multiplied by 1.6449. The African region definition is shown in Fig. S2.

Some remote impacts of the changed emissions are visible, with southern Europe and the Middle East exhibiting consistent changes with those found in North Africa. The impact in each scenario relative to the control on global and African PM_2.5_ and O_3_ annual deaths in 2090 is shown in Table 2, and the total deaths are shown in Fig. 3 over Africa and globally, with additional regions in the Supplement. Overall, Africa following SSP370 rather than SSP119 emissions leads to around 150 000 (5th–95th confidence interval of 67 000– 234 000) additional annual deaths across Africa from PM_2.5_ and 15 000 (5th–95th confidence interval of 9000–21 000) from O_3_ when the background populations are held constant. In Africa, air pollutant trends are consistent with the impact changes (Fig. S3). O_3_ is projected to approximately match PM_2.5_ in its health impacts by 2090 in these scenarios, due to the weaker decline in O_3_ levels than in PM_2.5_ (Fig. 3). The short pollutant lifetime, coupled with internal variability, causes the impacts outside Africa to be noisier than those within the continent; note that only 1 year (2090) was used for the analysis, as discussed in Sect. 2.3.

## 4 Discussion and conclusions

This study used the Earth system model UKESM1 to explore the range of impacts from future African pollutant emissions on air quality and premature mortality. Compared to SSP119, SSP370 has much higher fossil fuel and biofuel emissions but lower African biomass burning emissions; the reasons for this are unclear and reflect methodological challenges within the SSP framework. The increase in non-biomass emissions far outweighs the decrease in biomass emissions, particularly over population centres. To evaluate the human health impacts of the future emissions, CRFs were used from the recent GBD2019 study (GBD 2019 Risk Factors Collaborators, 2020) for six COD for PM_2.5_ and Turner et al. (2016) for O_3_ respiratory impacts. Estimates were calculated using present-day demographics to isolate the effect of changes in pollutants on a given population.

The methodology of this study is not directly comparable to comprehensive estimates of the present-day impact of air pollution, since only one model with no bias correction is used here; the focus of the analysis is on the differences between scenarios instead. However, the magnitude of the estimated impacts can be contextualised against prior studies to explore the effect of these methodological differences. Our estimate for total PM_2.5_-related deaths in 2015, of 2.76 (2.11–3.48) million, is generally lower than prior studies; its central estimate is lower than some (Lelieveld et al., 2019; Vohra et al., 2021; IHME, 2020; Im et al., 2023; Bauer et al., 2019) and higher than at least one other (Partanen et al., 2018). GBD2019 found higher impacts than those found here, using the same CRFs but different PM_2.5_ concentrations, and also including neonatal deaths. The dominance of the uncertainties in CRF over those in PM_2.5_ concentrations is consistent with prior research (Shindell et al., 2018).

The number of present-day respiratory deaths attributed here to O_3_ exposure (2.28 (1.73–2.70) million) is generally higher than found in previous studies, e.g. Anenberg et al. (2010) and Silva et al. (2016), which both used an earlier CRF reflecting weaker associations between O_3_ and health impacts (Jerrett et al., 2009) than that used here. Studies utilising the CRFs used in this study (Turner et al., 2016) also find lower numbers of deaths than estimated here (Shindell et al., 2018; GBD 2019 Risk Factors Collaborators, 2020; Malley et al., 2017), likely due to higher O_3_ concentrations in UKESM1 (see Sect. 2.1), but sparse observations preclude a full evaluation. The estimated O_3_ impacts were not very sensitive to changes in the LCT. The uncertainty in deaths due to the uncertainty within each CRF (Table 1) is far larger than that from intra-ensemble pollutant variations for both PM_2.5_ and O_3_, consistent with prior studies determining the CRF to be the largest source of uncertainty (Turnock et al., 2016; Johnston et al., 2012; Li et al., 2016; Shindell et al., 2018).

The effect of Africa following SSP370 rather than SSP119 is estimated to result in 150 000 additional annual deaths from PM_2.5_ and 15 000 from O_3_, across Africa in 2090, when using 2015 populations. Due to the large decrease in aerosol emissions in SSP119, annual PM_2.5_ deaths could be similar to those due to O_3_ by the end of the century. However, this result may also be affected by the O_3_ biases.

Correcting for model biases in PM_2.5_ and O_3_ concentrations in a CMIP6 model with low-biased concentrations was found to substantially affect estimated health impacts (Im et al., 2023), though this effect was strongest over high-emission regions and dampened when using more recent non-linear CRFs. The results of the scenarios in this study should therefore be primarily interpreted relative to each other, as the substantial differences between scenarios are less affected by the model biases.

Present-day populations were used in this study to isolate the effect of changes in air pollutants alone and because of the difficulties in projecting changes in mortality rates. Increasing and ageing future populations will lead to higher estimated deaths and hence larger reductions in deaths upon emission mitigation. In SSP1, the present-day African population of 1 billion increases to around 1.7 bn by 2070, before declining slightly; in SSP3, it increases throughout the century, reaching 4 bn by 2090 (Lutz et al., 2018). Projected urbanisation (Jiang and O’Neill, 2017) will increase the co-location of population centres and emissions, increasing the human health impacts of air pollution (Silva et al., 2017). This co-location is already dampened by the coarsemodel grid, which reduces the estimated impacts (Li et al., 2016; Likhvar et al., 2015), an effect which will be more pronounced for PM_2.5_ than for ozone (Malley et al., 2017). Prior work has found changes in populations play a comparable role to those in emissions in the SSPs (Im et al., 2023), though this estimate assumed the persistence of present-day baseline mortality rates.

Relatively higher non-BB aerosol south of the Sahara weakened the local surface circulation, reducing dust emissions sufficiently to reduce overall PM_2.5_ levels (and hence deaths) in some areas, demonstrating the importance of accounting for natural aerosols and circulation impacts when estimating the impacts of emission changes (Bauer et al., 2019; Yang et al., 2017). This effect will likely vary substantially between models, due to differing aerosol impacts and parameterisations of dust emissions.

The CRFs used in this study are generated by combining multiple cohort studies (GBD 2019 Risk Factors Collaborators, 2020). The more recent CRFs used here cover a wider range of air pollutant concentrations than those in earlier studies, but there are still limitations in the representativeness of the input data used to generate the CRFs, stemming from structural and historic challenges in air pollution research in Africa (Abera et al., 2021; Katoto et al., 2019; Coker and Kizito, 2018; Pinder et al., 2019). Further research characterising appropriate CRFs for use in disparate regions is essential to generate more reliable estimates of air pollution impacts. If BC has a higher toxicity (Lelieveld et al., 2015; Coker and Kizito, 2018), future air pollutant impacts per unit change in PM_2.5_ concentrations would be reduced as the BC share of PM_2.5_ declines and the effect of PM_2.5_ mitigation therefore enhanced.

UKESM1’s horizontal resolution is coarser than the relevant scales for localised air pollutants from different sources, as the distinction between rural/urban and emissions from vehicles, factories, and domestic fuel is dampened by averaging across the model grid cells. Global models are incapable of resolving these distinctions, which are of relevance for policy and behavioural considerations. Models of this resolution still clearly resolve distinctions between high- and low-air-pollutant regions (Fig. 1), and this method has been applied in many prior studies, often at coarser resolutions, to generate understandings of the global mortality impact of air pollution (Shindell et al., 2018; Lelieveld et al., 2019, 2013; Vohra et al., 2021; Silva et al., 2016; Partanen et al., 2018; Anenberg et al., 2010; Silva et al., 2017).

Pollutant concentrations from single years were used to estimate the health impacts (see Sect. 2). While the intra-ensemble mean was used, the variation in the concentrations manifests in large variations in the projected impacts over heavily populated regions, which had no emission change in our experiments, such as in Asia.

The effect of different future African emission pathways on human health is large; reductions in the anthropogenic African aerosol emissions through climate mitigation within the range of the SSPs can reduce annual deaths by 150 000 for PM_2.5_ and 15 000 for O_3_, compared to a more polluted pathway, using present-day demographics. These values can be expected to be larger under future increasing and ageing populations. These results are focused on 2090, but the rapid emission drop in SSP119 suggests that significant benefits would occur much faster under such a scenario. Substantial near-term localised reductions in the impacts of air pollution could therefore be obtained as co-benefits of climate change mitigation in Africa.

## Supplementary Material

Supplement

## Figures and Tables

**Table 1:** Annual deaths (in thousands) in 2015 attributable to PM_2.5_, shown globally in total and for each COD separately, and the total across several regions (definitions in Fig. S2) and those attributable to respiratory illnesses caused by O_3_ exposure globally and in several regions. Also shown is the effect of using a low-concentration threshold (LCT, above which no harm is assumed; see Sect. 2) of 31.1 ppb on global deaths for O_3_, instead of the 26.7 ppb used for the main results. Values are shown using the central CRF estimate and the 5th and 95th percentile CRF estimates, all from GBD2019 for PM_2.5_ and Turner et al. (2016) for O_3_. The uncertainty given for each estimate is the estimated 5th–95th percentile range across the 10 control ensemble members, calculated as the standard deviation multiplied by 1.6449. The region definitions are shown in Fig. S2.

Species	Region	COD	Middle RR (thousands of deaths yr^−1^)	Low RR (thousands of deaths yr^−1^)	High RR (thousands of deaths yr^−1^)
PM_2.5_	Global	All	2760 ± 30	2110±30	3480±30
		COPD	310 ± 4	245 ± 4	377 ± 4
		IHD	920 ± 10	697 ± 9	1190 ± 10
		LC	196 ± 3	148 ± 3	247 ± 3
		Stroke	870 ± 10	690 ± 10	1060±10
		T2DM	197 ± 1	145 ± 1	254 ± 1
		LRI	264 ± 6	184 ± 5	361 ± 8
PM_2.5_	Africa	All	340 ± 10	250 ± 10	430 ± 10
	Europe		58 ± 6	22 ± 3	111 ± 9
	Asia		2180±30	1730 ± 20	2660 ±30
	West Africa		121 ± 9	93 ± 8	150 ± 10
O_3_	Global	Respiratory	2280 ± 30	1730±30	2700 ± 30
	Global 31.1 ppb		2160±30	1630±30	2570 ± 40
	Africa		169 ± 3	125 ± 3	204 ± 4
	Europe		139 ± 5	100 ± 3	172 ± 5
	Asia		1680±30	1290±20	1970±30
	West Africa		46 ± 1	34 ± 1	55 ± 1

**Table 2:** Effect of each scenario, relative to the control, in thousands of annual PM_2.5_ and O_3_ deaths in 2090 globally and just over Africa, using 2015 populations and the central CRF. Values are bold when they are more than 1 intra-ensemble standard deviation away from 0. The African region definition is shown in Fig. S2.

Experiment	PM _2.5_ (thousands of deaths in 2090)		O_3_ (thousands of deaths in 2090)	
Global	Africa	Global	Africa
AerNonBB	**130**	**154**	**23**	**24**
AerBB	**− 44**	−2	**− 22**	**−18**
AerAll	**151**	**152**	**25**	**15**

## Data Availability

Reasonable requests for model output and the data used for figures in this article can be made to the corresponding author.
