# Supplementary material for: Significant human health co-benefits of mitigating African emissions
Source: Atmos Chem Phys. Author manuscript; Available in PMC 2024 Feb 12. (PMC7615628; doi:10.5194/acp-24-1025-2024)
Supplement: Supplement [file EMS193617-supplement-Supplement.pdf]

Supplement of Atmos. Chem. Phys., 24, 1025–1039, 2024
https://doi.org/10.5194/acp-24-1025-2024-supplement
© Author(s) 2024. CC BY 4.0 License.

Atmospheric Chemistry and Physics
Open Access

Supplement of

Significant human health co-benefits of mitigating African emissions

Christopher D. Wells et al.

Correspondence to: Christopher D. Wells (c.d.wells@leeds.ac.uk)

The copyright of individual parts of the supplement might differ from the article licence.

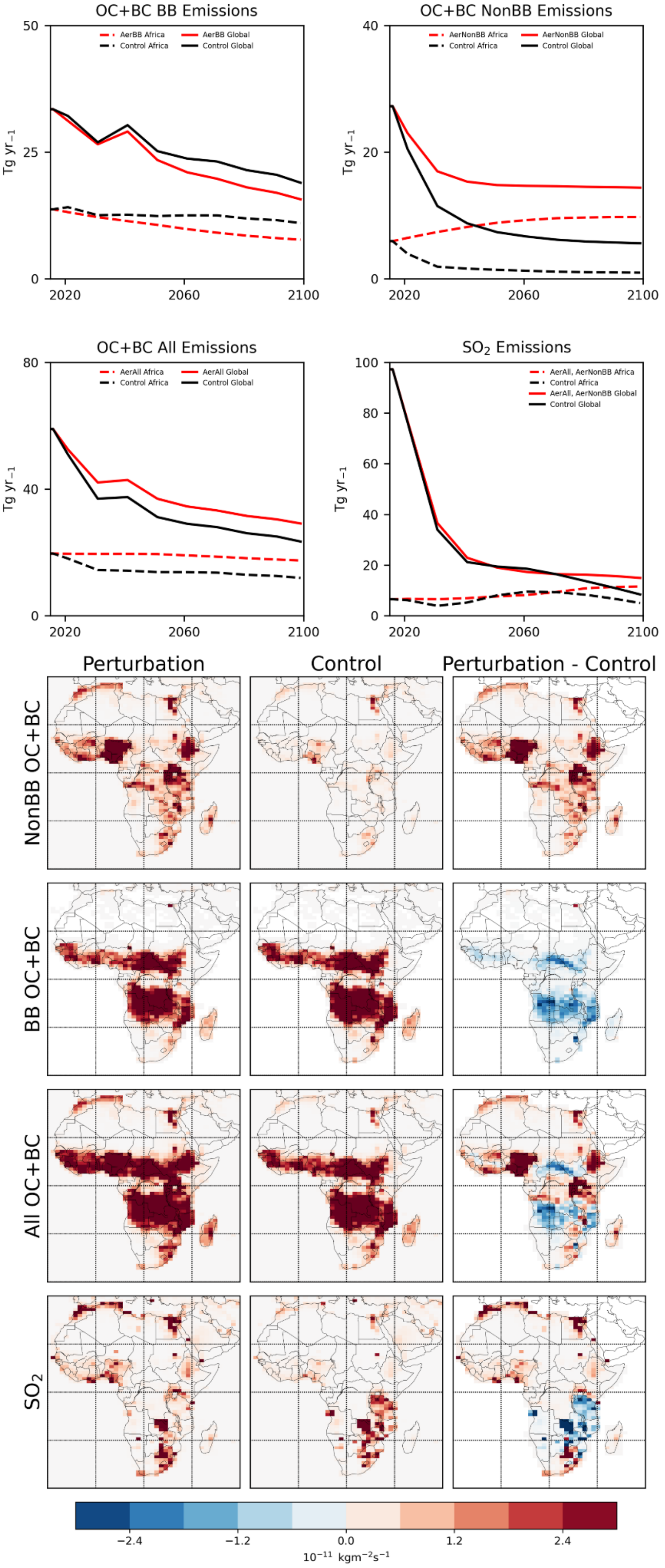

OC+BC BB Emissions
AerBB Africa
AerBB Global
Control Africa
Control Global
Tg yr−1
OC+BC NonBB Emissions
AerNonBB Africa
AerNonBB Global
Control Africa
Control Global
OC+BC All Emissions
AerAll Africa
AerAll Global
Control Africa
Control Global
SO2 Emissions
AerAll, AerNonBB Africa
Control Africa
AerAll, AerNonBB Global
Control Global
Perturbation
Control
Perturbation - Control
NonBB OC+BC
BB OC+BC
All OC+BC
SO2
10−11 kgm−2s−1

Figure S1: Timeseries of total and BB carbonaceous aerosol and SO_2 emissions in the control (black) and experiments (red), globally (solid lines) and over Africa (dashed lines). The red lines in the OC+BC total plot therefore show the AerAll emissions, while the red line in OC+BC BB shows those of AerBB. Also shown are maps of the emissions changes over Africa in each experiment. The Africa region definition is shown in Figure S2. This figure is similar to – and adapted from – Figure 1 of Wells et al 2023 https://doi.org/10.5194/acp-23-3575-2023.

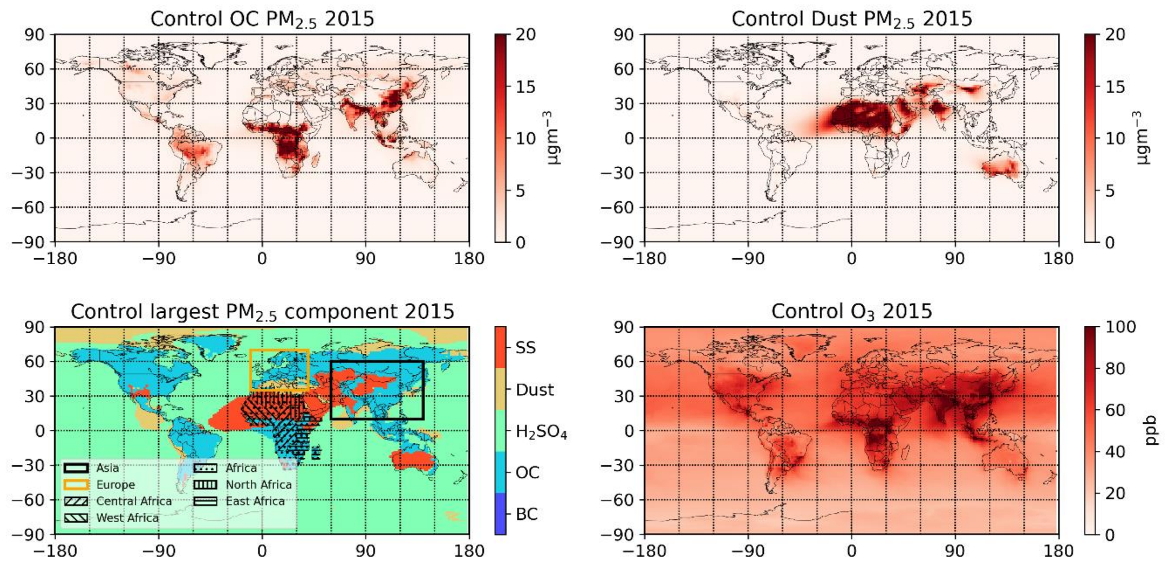

Figure S2: OC and dust contributions to surface $PM_{2.5}$, and surface O_3 concentrations, in the control in 2015. Also shown is the largest component of $PM_{2.5}$ in each gridcell; region definitions for Table 1 and Figures 3, S1, and S3 are also shown in this panel.

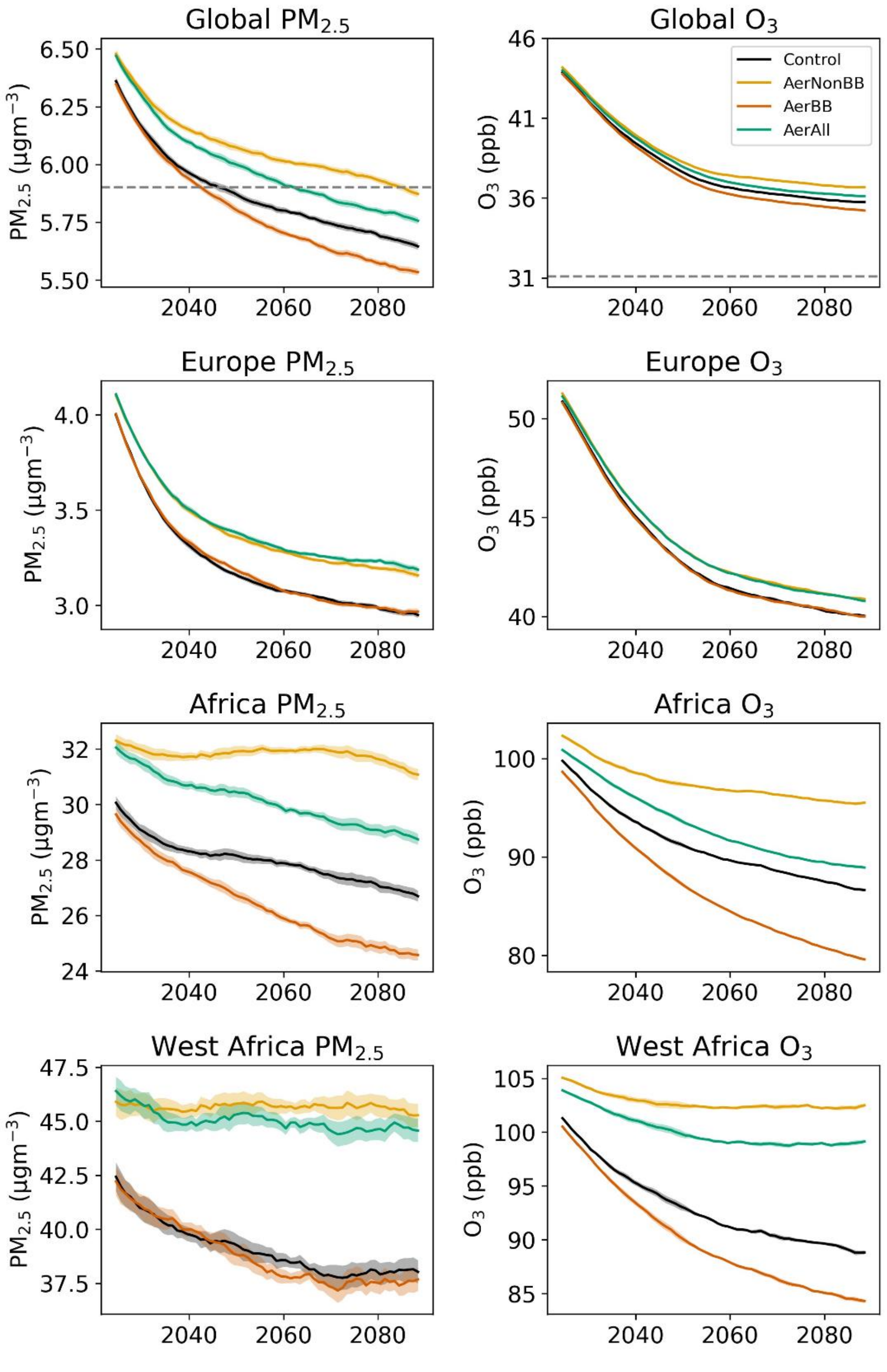

Figure S3: Timeseries of surface $PM_{2.5}$ and O_3 concentrations in each experiment, globally and in several regions. The region definitions are shown in Supplementary Figure S2. The timeseries are 20-year rolling means of annual averages. The dotted line on the global $PM_{2.5}$ plot indicates 5.9µg m^{-3}, the upper boundary of the LCT; the dotted line on the global O_3 panel shows the 31.1ppb LCT used in the high-LCT sensitivity test, indicating that global concentrations lie above the LCT in all experiments. The region definitions are shown in Figure S2.

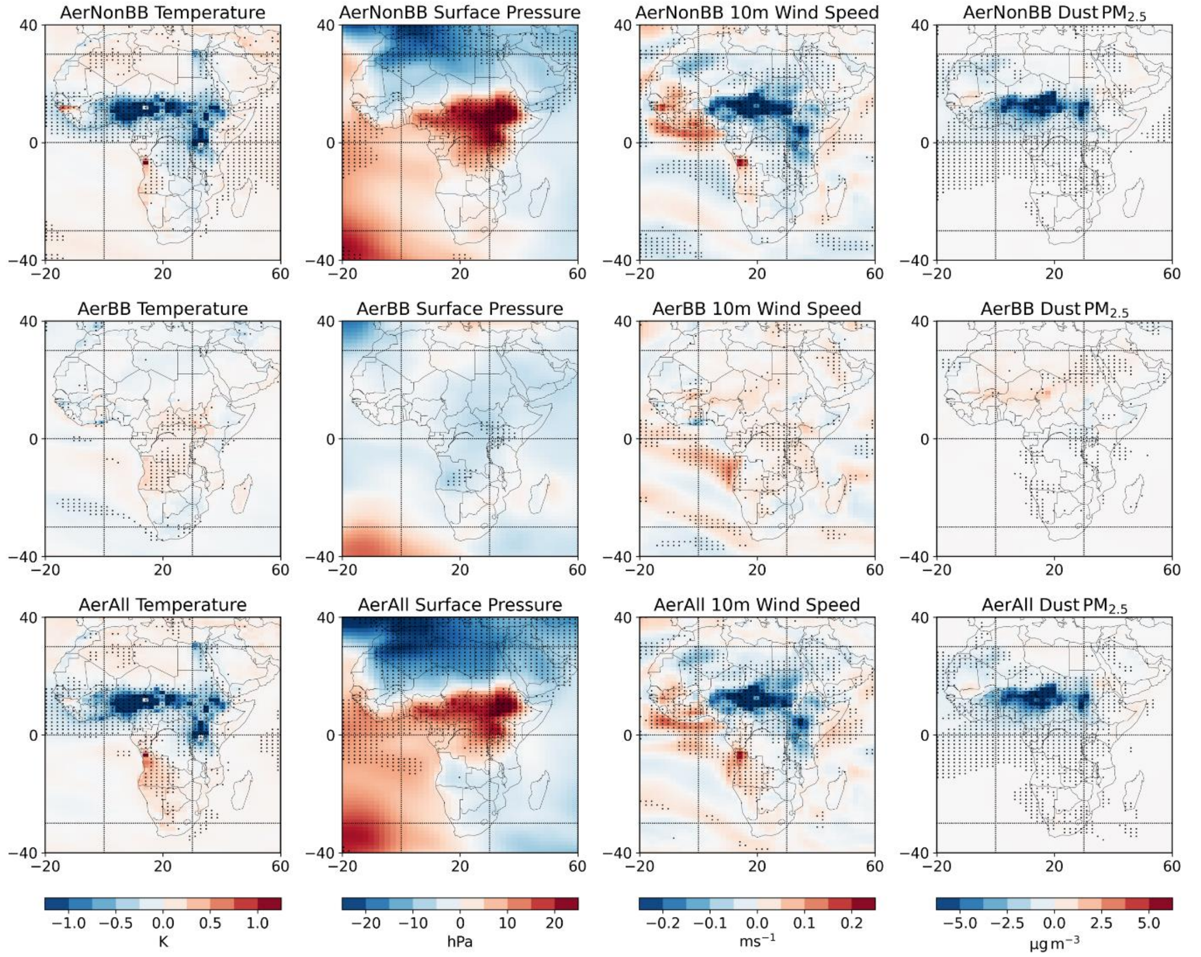

Figure S4: 2070-2100 changes in surface temperature, pressure, wind speed (at 10m), and dust PM$_{2.5}$ contribution over Africa under each scenario. Stippling indicates areas where the change is statistically significant, defined as being greater than one intra-ensemble standard deviation away from zero. As shown in Figure 1, the dust contribution to PM$_{2.5}$ decreases under increased non-BB emissions over parts of the southern Sahara. This reduces overall PM$_{2.5}$ in some regions away from the central areas with increased anthropogenic emissions, acting to reduce PM$_{2.5}$-attributable deaths across a band of northern tropical Africa (Figure 6), despite the increase in anthropogenic emissions. This reduction in dust aerosol is a result of circulation changes induced by the strong emissions increases under SSP370 non-BB aerosol emissions relative to SSP119. Figure S4 shows the effect of each scenario on surface temperature, pressure, wind speed (calculated at 10m), and dust PM$_{2.5}$, averaged across 2070-2100. The enhanced aerosol generates a strong cooling response over northern tropical Africa, due to the reduced radiation incident to the surface. This causes local stagnation and reduced wind speed, and hence reduced dust emissions. While dust PM$_{2.5}$ is decreased across a broad area, it is outweighed in the total PM$_{2.5}$ – and hence PM$_{2.5}$-attributable deaths – by the increases in anthropogenic emissions which induced the change. But further from the core emissions regions, e.g. in southern Chad, the decreased dust dominates, reducing PM$_{2.5}$, and hence PM$_{2.5}$-linked deaths.

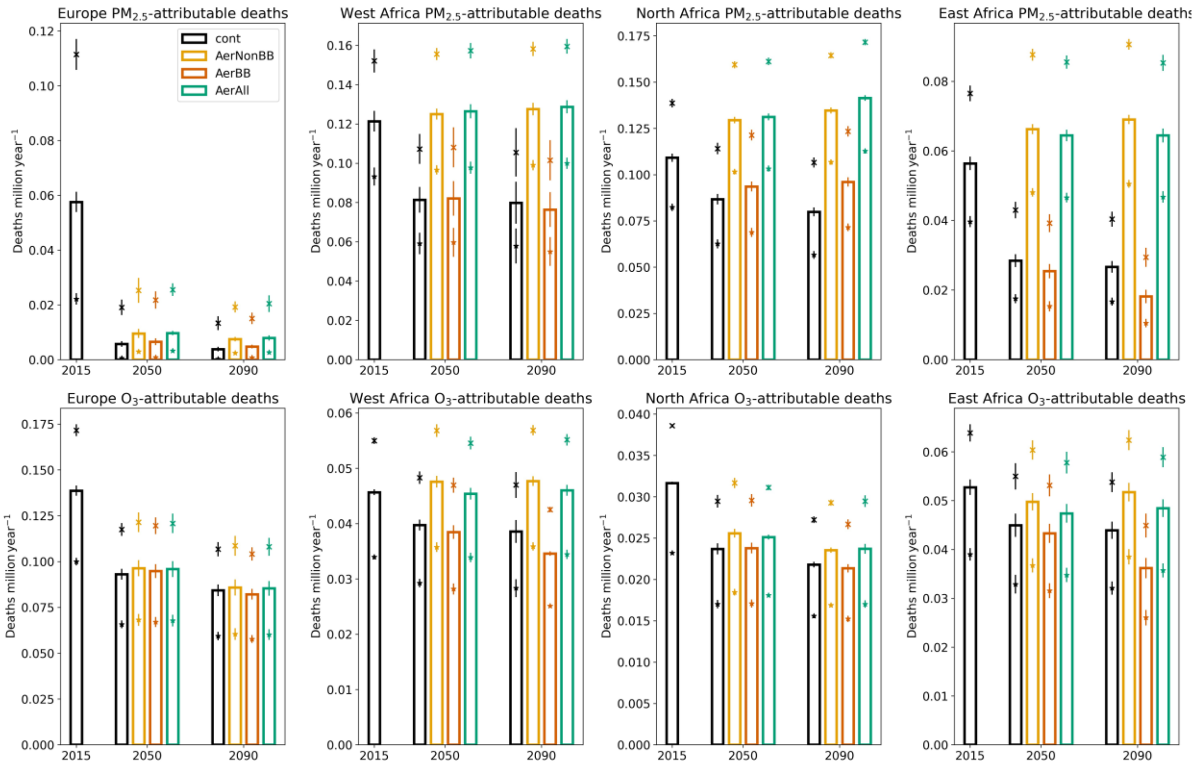

Figure S5: Annual $PM_{2.5}$- and O_3-attributable deaths in the control in 2015, and each scenario in 2050 and 2090, over several regions. The bars indicate the estimated deaths using the central CRF estimates; the crosses and stars use the 95th and 5th percentile CRF values respectively. For each CRF value, the (much smaller) uncertainty due to intra-ensemble variation in pollutant concentrations is indicated, with horizontal bars indicating the mean and plus and minus one standard deviation. The region definitions are shown in Figure S2.
